# Influence of Water Exposure on the Relaxation Behaviour of Short Fibre Reinforced Polycarbonate

**DOI:** 10.3390/polym18151795

**Published:** 2026-07-23

**Authors:** Pean-Yue Ben Jar, Jingchao Wang

**Affiliations:** Department of Mechanical Engineering, University of Alberta, Edmonton, AB T6G 1H9, Canada

**Keywords:** short glass fibre, polycarbonate, hydrothermal treatment, mechanical testing, spring-dashpot modelling

## Abstract

An approach based on a multi-relaxation (MR) test, in conjunction with a spring-dashpot model for data analysis, was employed to evaluate the influence of hydrothermal treatment at 65 °C on the viscous and quasi-static stress responses of polycarbonate (pure PC) and its short glass fibre composite (GF-PC) during the relaxation stages. The findings indicate that while this hydrothermal treatment did not affect the ductility of pure PC, it resulted in a significant ductility decrease in GF-PC. Furthermore, results from the modelling indicated that a parallel three-branch model was sufficient to simulate the stress decay during the relaxation stages of pure PC, but an additional branch (designated as the M-branch) was required for GF-PC. The study also found that for GF-PC, the hydrothermal treatment led to a distinct increase in the viscous stress response of the M-branch, but not in the other two viscous branches. This phenomenon is believed to stem from the coexistence of two types of PC matrix in GF-PC: one influenced by the presence of fibres and the other unaffected. Without the hydrothermal treatment, both types of PC matrix exhibit a similar deformation-dependence (based on stroke of the test machine) in their stress response; however, because the hydrothermal treatment affects the strength at the fibre–matrix interface, the stress responses of the two matrix types differ during relaxation. Consequently, the M-branch is believed to capture the altered stress response of the fibre-influenced PC matrix. The study concludes that under the investigated hydrothermal conditions, the reinforcing effect of the short fibre on the mechanical strength of GF-PC could be lost. Therefore, the addition of short glass fibre does not inherently guarantee mechanical reinforcement of PC.

## 1. Introduction

Work described here is a follow-up to a previous study [1] in which an approach based on a multi-relaxation (MR) test [2], along with a parallel three-branch spring-dashpot model for data analysis, was utilized to characterize the mechanical properties of pure polycarbonate (PC) and its short glass fibre reinforced composite (GF-PC). Our early work [3,4,5] has demonstrated two major advantages of this approach over the traditional mechanical testing. One is the ability to decouple the time-dependent stress response to deformation from the time-independent counterpart (i.e., the viscous and quasi-static components, respectively); the other is the capacity to generate multiple datasets from a single test, whereas the traditional testing provides only one dataset per test. However, for the material system of PC and GF-PC, the previous study identified certain limitations for this approach [1]. Specifically, the three-branch model could not always provide a satisfactory fitting for the stress decay of GF-PC at the relaxation stages, particularly at deformation levels exceeding 30% of the yield strain. Consequently, the first task in the current work is to modify the spring-dashpot model to have the capability of replicating closely all stress decay curves of GF-PC. Then, the work is to examine whether the modified model has the ability to simulate stress decay at relaxation stages even after some mechanical property changes, thereby exploring the capability of this approach that has not yet been examined before.

In the current work, a hydrothermal treatment was used to change mechanical properties of GF-PC. Work in the literature has shown that a hydrothermal treatment could affect interfacial bonding in fibre composites [6,7,8]. Although the most popular approaches to examining the change in interfacial bonding between fibre and matrix are using micro-mechanical tests, such as pull-out [9,10,11], micro-bond [12,13], single-fibre fragmentation [14,15], and push-in or push-out [16,17,18] tests, to name a few, many factors are known to cause discrepancies between results from these tests and the bulk mechanical properties of the fibre composites, including sizing used on fibre surface [19], bead size of the polymer resin [20], the constraint conditions in the test [21,22], the strain rate applied in the test [23,24], and the loading mode [25]. While some modifications have been proposed for these tests to be able to closely mimic the fracture environment of the bulk material [26,27], the test results may still show inconsistency with those from bulk materials. Therefore, rather than the micro-mechanical tests, the work presented in this paper uses MR test on bulk specimens to evaluate the efficacy of fibre on the enhancement of mechanical properties of the composites.

The composite material chosen for this study is GF-PC, leveraging the experimental conditions and analytical framework established in our previous study [1]. To induce changes in mechanical properties, a hydrothermal treatment protocol based on immersion in water was developed following an extensive literature review covering various polymers and composites with distinct fibre–matrix interfaces [28,29,30,31,32,33,34,35,36,37]. Although only limited data are available for the influence of temperature and immersion duration for the hydrothermal treatment on GF-PC, a general trend emerges from the literature: lower temperatures reduce changes in the inherent properties of the polymer matrix. Consequently, a relatively low temperature of 65 °C was selected for this study, which is below the temperatures typically applied to PC in previous investigations [30,32,35,37]. To effectively target the changes in the interfacial strength, a prolonged treatment duration exceeding 1000 h was implemented, which allows the specimen weight gain to reach a plateau prior to MR testing.

Because the exact mechanisms driving the hydrothermal degradation of mechanical properties are highly time- and temperature-dependent—and because detailed material characteristics (such as molecular weight and distribution of the commercial PC) were unavailable to us—pure PC specimens with identical in-plane dimensions and similar thicknesses were prepared. Subjected to the same hydrothermal treatment, these pure PC specimens served as a baseline reference to determine if the PC matrix itself underwent standalone degradation in the hydrothermal treatment. Any divergence in the mechanical behaviour of GF-PC from this parallel trend of pure-PC could then be related to the changes at the fibre–matrix interface—a phenomenon of interest to the research community. Ultimately, by utilizing the pure PC data to rule out the hydrothermal degradation within the matrix, the objective of this study is to determine whether the combination of MR testing and spring-dashpot modelling can be effectively used to characterize GF-PC and isolate the interfacial effects from the matrix degradation.

## 2. Experimental Details and Modelling Approach

### 2.1. Experimental Details

#### 2.1.1. Dog-Bone Specimens

**Figure 1:**
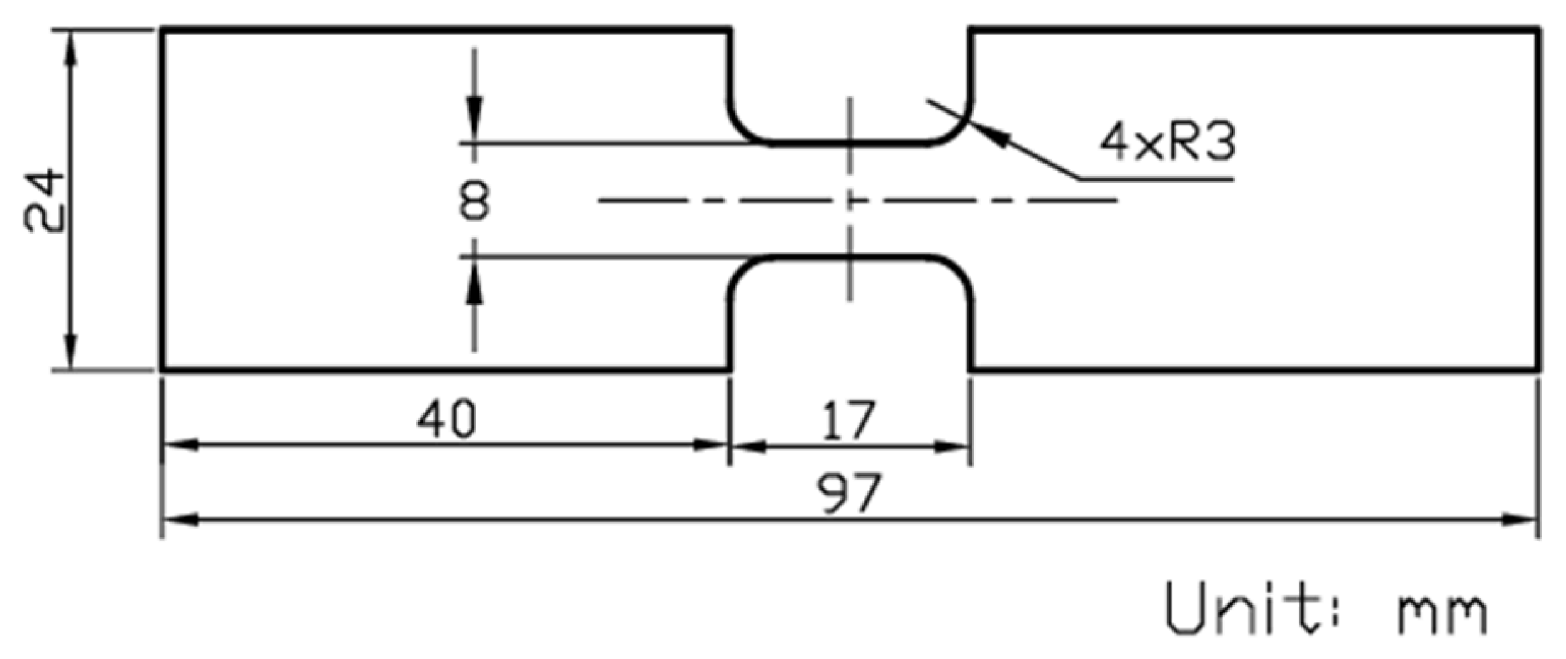
Dimensions of specimens used in the MR test.

Dog-bone specimens were water-jet cut from pure PC and GF-PC plaques obtained from McMaster-Carr (Santa Fe Springs, CA, USA), with the GF-PC plaques containing 20 wt% short glass fibre. The thicknesses of the pure PC and GF-PC plaques were chosen to be close to each other: 9.3 mm and 10.5 mm, respectively. Figure 1 shows the in-plane dimensions of the dog-bone specimens.

#### 2.1.2. Hydrothermal Treatment

The hydrothermal treatment was conducted by placing water containers holding the specimens in a Fisher Scientific thermal chamber (Waltham, MA, USA), which was calibrated using a thermocouple to ensure the water temperature remained at 65 °C. The weight gain of the specimens was regularly monitored during the treatment to ensure that the MR tests were conducted only after water absorption had reached a saturation level, requiring a period of over 1000 h.

The hydrothermal treatment was applied to a minimum of three specimens for both pure PC and GF-PC: two were designated for the MR test, while the third was used to monitor weight change throughout the testing period.

#### 2.1.3. MR Test

The loading scheme of the MR tests conducted in this study was similar to that used previously [38,39], consisting of multiple loading stages at a crosshead speed of 1 mm/min, with an increase in specimen displacement at each loading stage by approximately 0.065 mm. After each loading stage, the crosshead stroke of the testing machine was held constant for 10,000 s to allow for stress relaxation. A Quasar 100 universal testing machine (by Cesare Galdabini, Cardano al Campo, Italy) was used to conduct all MR tests at ambient temperature. Tests on GF-PC were terminated automatically upon specimen fracture or a significant load drop, whereas tests on pure PC were terminated when, following the peak load, the maximum force applied at each loading stage remained constant for several loading–relaxation cycles, which occurred at a stroke of at least 3 mm.

Given that the pure PC specimens served as a reference to evaluate the effect of water absorption on the matrix of GF-PC, data analysis for the pure PC was conducted up to a stroke of approximately 1.7 mm, corresponding to the point at which fracture or a significant load drop of the GF-PC specimens.

**Figure 2:**
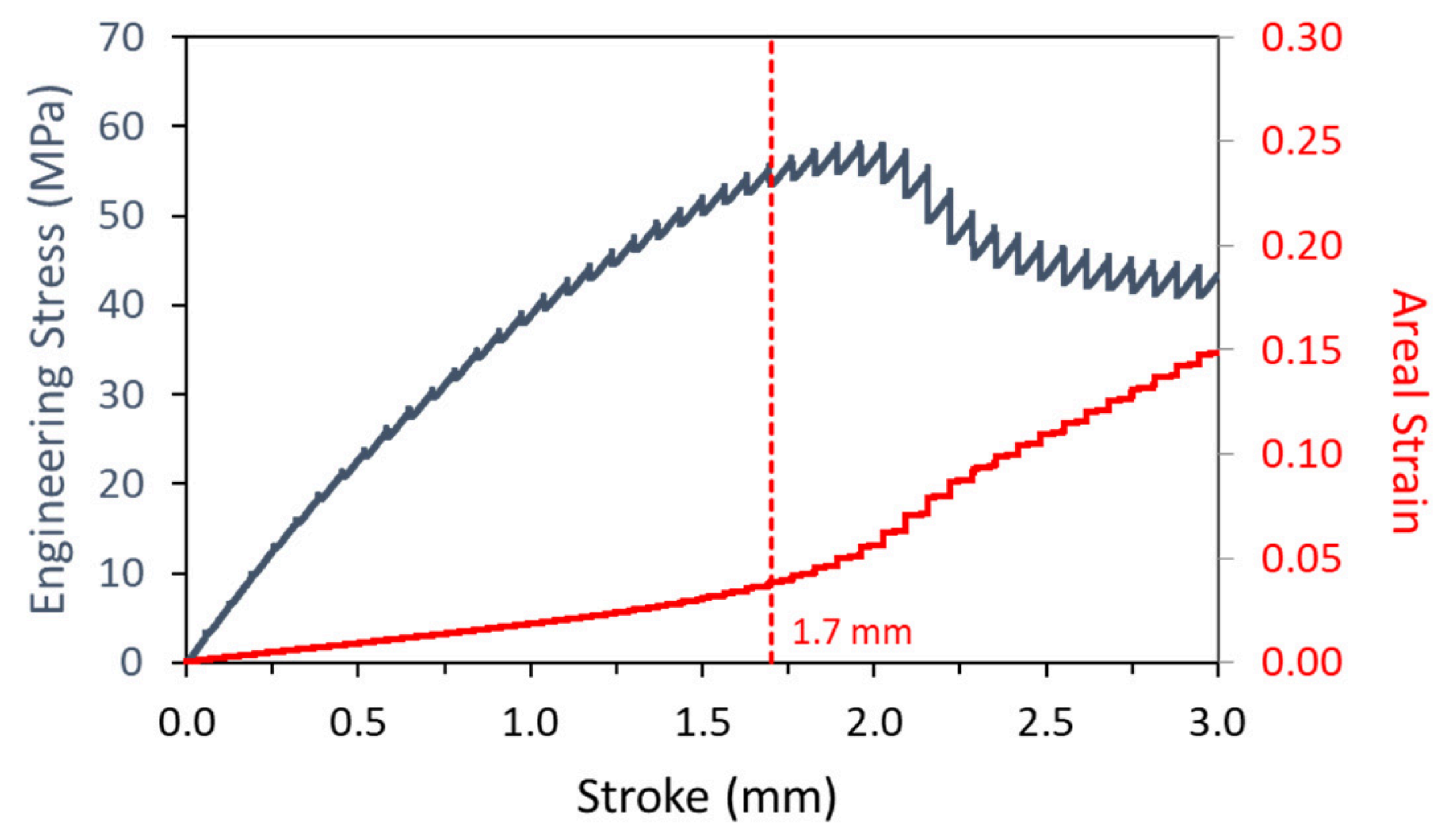
Sample curves from a pure PC specimen showing engineering stress and areal strain as functions of stroke at a crosshead speed of 1 mm/min.

An extensometer was mounted to the middle of the gauge section to record width changes during testing. These measurements were then used to calculate the areal strain, defined as $2\times ln\left(W_{o}/W_{d}\right)$, where $W_{o}$ is the original width in the gauge section and $W_{d}$ is the width of the deformed gauge section at the same location. Figure 2 shows sample data for a pure PC specimen, including engineering stress and areal strain as functions of stroke. The figure demonstrates that up to a stroke of 1.7 mm, the areal strain and stroke followed a monotonic relationship, with negligible change in areal strain during relaxation. Therefore, the stroke is used hereafter to represent the deformation of the specimens.

It is known that traditional mechanical testing requires a minimum of three replicates for unnotched specimens and five replicates for notched specimens to ensure statistical reliability. For the MR test, however, two specimens suffice for this purpose. In view that each MR test incorporates multiple consecutive loading–relaxation stages, a single specimen produces a volume of data equivalent to approximately 25 traditional individual tests. Furthermore, as demonstrated in Section 3 ‘Test Results and Discussion,’ the two specimens produced highly reproducible, overlapping curves up to a stroke of 1.7 mm. Therefore, testing two specimens should be sufficient for the analysis using the spring-dashpot model.

### 2.2. Spring-Dashpot Models

**Figure 3:**
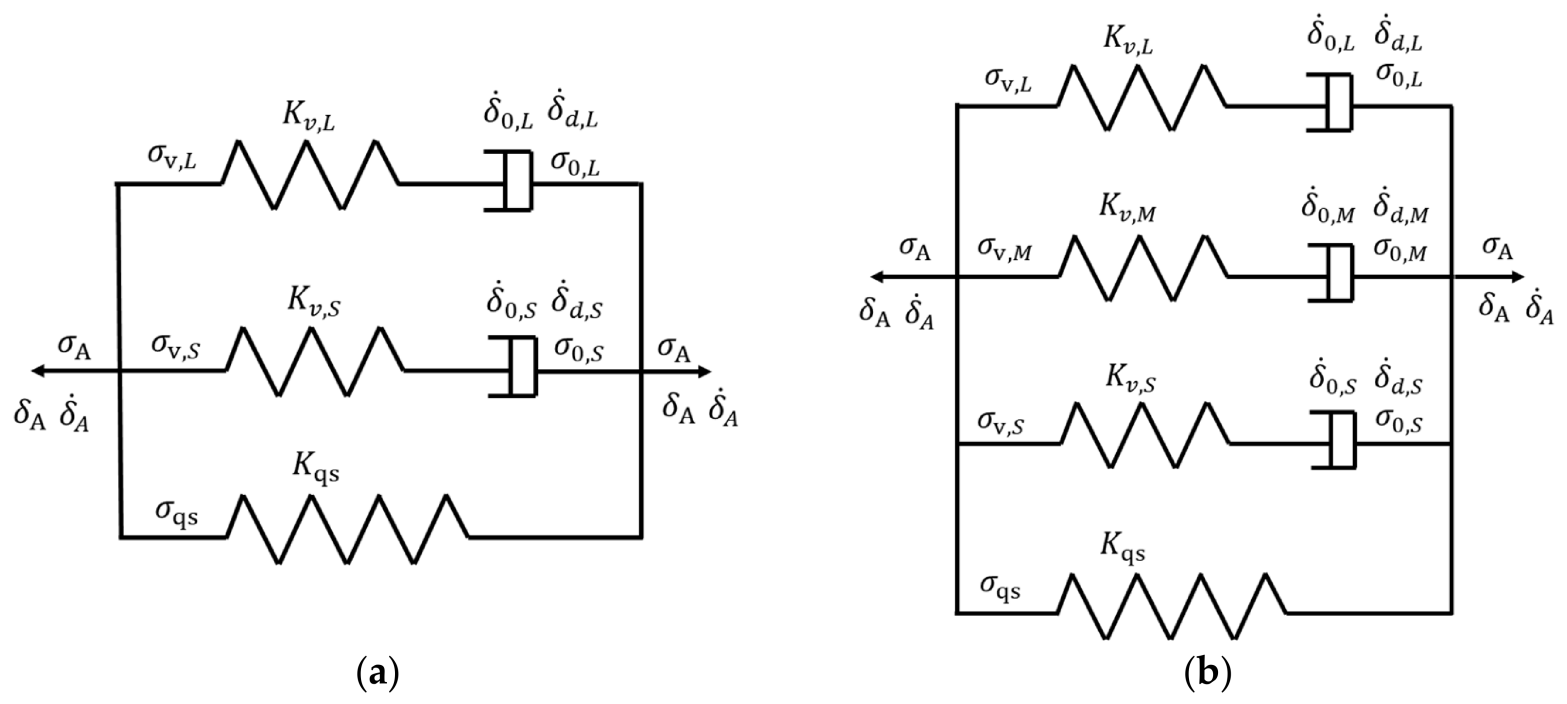
Spring-dashpot models used in this study: (**a**) the three-branch model for pure PC and (**b**) the four-branch model for GF-PC.

Following previous work [1], a parallel three-branch model was used to simulate the stress decay during the relaxation stages of the pure PC specimens, as shown in Figure 3a. However, that study demonstrated that for GF-PC, the three-branch model failed to provide a satisfactory fit to the stress decay within the first 10 s of the relaxation stages at specimen displacements up to 0.72 mm, whereas the ultimate fracture displacement was only about 1.7 mm. Consequently, a preliminary optimization study was conducted to determine the minimum number of branches required in a spring-dashpot model to accurately capture the relaxation behaviour of GF-PC.

Initially, a five-branch model—comprising one quasi-static branch, two long-term (L) branches, and two short-term (S) branches—was found to be able to closely replicate the stress decay curves across all relaxation stages. However, the extracted parameter values for the two L-branches exhibited nearly identical patterns of variation as a function of specimen displacement. Since these two branches were mathematically redundant, they were merged into a single L-branch. This reduction yielded the four-branch model shown in Figure 3b, in which one of the original S-branches is designated as the intermediate (M) branch due to its relatively larger characteristic relaxation time.

Similar to the approach used previously [1], the stress responses ($\sigma_{v,i}$) of the dashpots in the models were governed by the Eyring Law [40], i.e.,

(1) $$\sigma_{v,i}/\sigma_{0,i}={sinh}^{-1}\left({\dot{\delta }}_{d,i}/{\dot{\delta }}_{0,i}\right)$$

where $\sigma_{0,i}$, ${\dot{\delta }}_{d,i}$ and ${\dot{\delta }}_{0,i}$ are the reference stress, the applied stroke rate, and the reference stroke rate of the dashpot in viscous branch *i*, with *i* = S, M, and L. ${\dot{\delta }}_{0,i}$ can be expressed using the following function:

(2) $${\dot{\delta }}_{0,i}=\sigma_{0,i}/\left(\tau_{v,i}\;K_{v,i}\right)$$

where $K_{v,i}$ and $\tau_{v,i}$ are the spring modulus and characteristic relaxation time, respectively, of viscous branch *i*.

**Figure 4:**
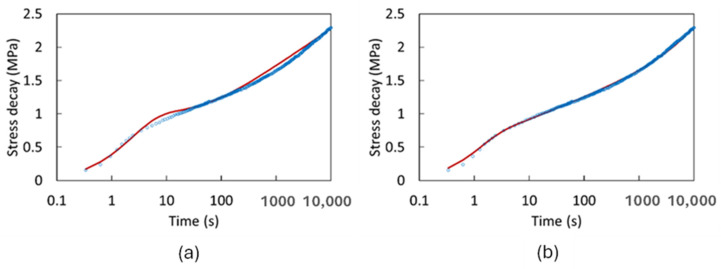
An example of the best-fitting curves (red) generated from (**a**) a three-branch model and (**b**) a four-branch model, compared with experimentally measured data (blue) for a GF-PC specimen at a stroke of 0.716 mm.

Figure 4 illustrates the difference between the three- and four-branch models when fitting a stress decay curve for a GF-PC specimen at a stroke of 0.716 mm.

### 2.3. Data Analysis

The data analysis utilized a genetic algorithm (GA), based on the principle of natural selection in biological evolution [41], to determine the fitting parameters required for the models to replicate the stress decay curves obtained from the MR tests. This fitting process presented two major challenges. One was the possibility of obtaining multiple sets of parameter values with significant variation but all could generate a similar fitting quality. The other was defining an appropriate search scope for the parameters, as this scope had to be broad enough to capture the optimal values, yet sufficiently narrow to ensure the parameters revealed clear trends across the entire specimen deformation range used in the MR testing, allowing them to effectively characterize the microstructural changes in the specimens.

For each relaxation phase, GA was executed to generate a population of 1000 candidate solutions over 60 generations. The crossover and mutation operators were governed by the default settings in MATLAB R2023b, which utilize scattered crossover and adaptive feasible mutation. The convergence criterion dictated that the optimization process was terminated either when the objective value—defined as the maximum absolute difference between the experimentally measured stress data and the fitted curve—fell below 0.08 MPa, or when the maximum number of generations was reached. Table 1 and Table 2 summarize the search scopes used in the current study for the three- and four-branch models, respectively.

From the 300 sets of parameter values, the 100 sets that yielded the lowest deviation from the experimental data were selected for subsequent analysis. In this study, the maximum deviation was found to be less than 0.071 MPa for the pure PC specimens and less than 0.064 MPa for the GF-PC specimens, encompassing both the untreated and hydrothermally treated conditions.

It should be noted that early work by Hong et al. [42] showed the feasibility of using the variation trend of the reference stress ($\sigma_{0}$) to determine critical deformation levels for microstructural changes in semi-crystalline polymers. However, they did not consider the possibility of multiple sets of $\sigma_{0}$ values yielding very similar fitting qualities. Our previous study [1] also demonstrated that when a broad search scope was used for $\tau_{v,S}$, the genetic algorithm could generate two distinct sets of $\sigma_{v,S}\left(0\right)$ and $\sigma_{v,L}\left(0\right)$ values, where the trend for $\sigma_{v,S}\left(0\right)$ in one set mirrored that of $\sigma_{v,L}\left(0\right)$ in the other set, and vice versa. In that case, we used the values of the corresponding spring moduli in the same viscous branches to demonstrate that one of the two parameter sets was merely a mathematical solution enabling the model to fit the experimental data, lacking any physical meaning regarding the microstructural changes in the material. Therefore, a search scope must be narrow enough to avoid duplicate parameter values that lack a meaningful physical interpretation. In addition, in view of a potentially wide range of fitting parameter values exist for a given set of experimental data, our discussion in this paper focuses mainly the range of stress variation for each branch of the spring-dashpot model, which at this stage is sufficiently consistent to draw some physically meaningful conclusions.

It should also be noted that to ensure consistency between the pure PC and GF-PC datasets, the search scope for $\tau_{v,S}$ in the four-branch model (Table 2) was made identical to that of the three-branch model (Table 1). This specific range—0 to 10 s—served to capture the first turning point observed in the stress decay curves of the GF-PC specimens. Similarly, the search scope for $\sigma_{0,L}$ in the four-branch model was set equal to that in the three-branch model (i.e., 0 to 3 MPa).

The governing equation for the total stress decay, $\Delta \sigma_{v}\left(t\right)$, is expressed as

(3) $${∆\sigma }_{v}\left(t\right)=\sum_{i}\sigma_{v,i}\left(0\right)-2\sigma_{0,i}{tanh}^{-1}\left[\tanh\left(\sigma_{v,i}\left(0\right)/2\sigma_{0,i}\right)\exp\left(-t/\tau_{v,i}\right)\right]$$

and the corresponding quasi-static stress ($\sigma_{qs}$) is defined as

(4) $$\sigma_{qs}=\sigma_{A}-\sum_{i}\sigma_{v,i}$$

where i = S, L for the three-branch model, and S, M, L for the four-branch model. Here, $\sigma_{v,i}$ is the viscous stress in the branch *i* as a function of time from the beginning of a given relaxation stage, and $\sigma_{v,i}\left(0\right)$ is the viscous stress at the start of a relaxation stage. The values of all these parameters are functions of specimen displacement, which is represented by the crosshead stroke of the testing machine.

## 3. Test Results and Discussion

### 3.1. Change in Water Content

**Figure 5:**
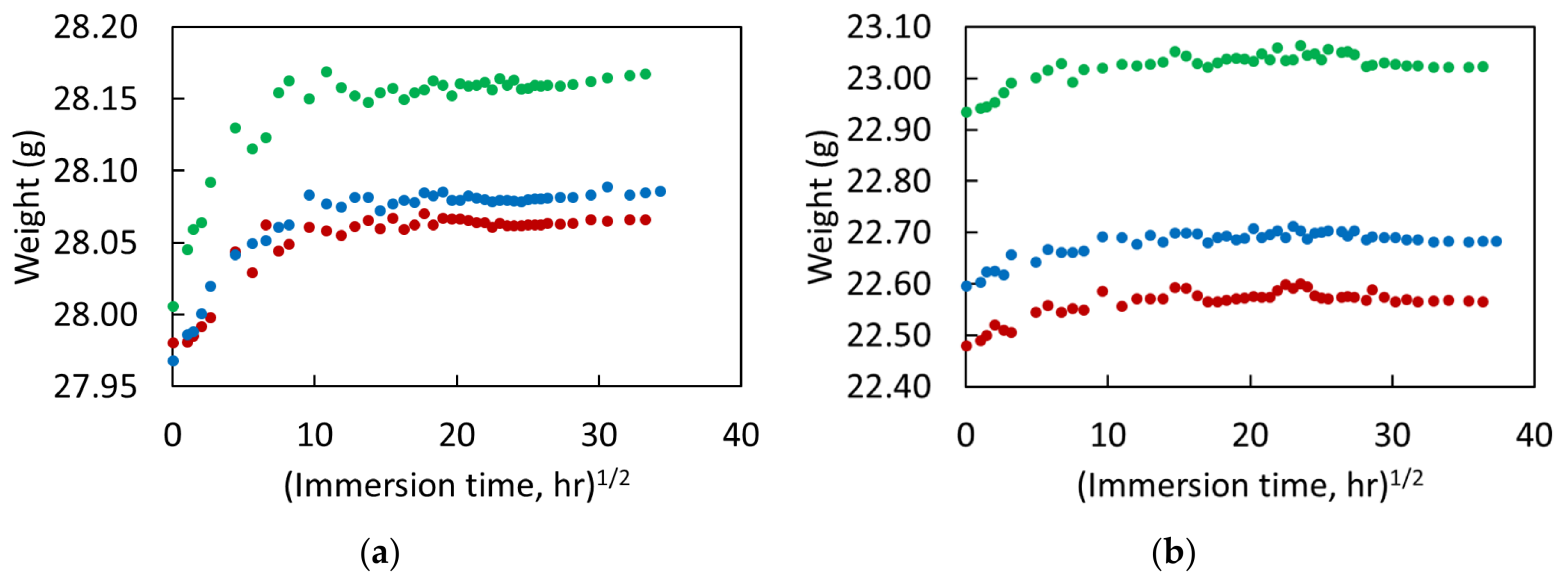
Weight change as a function of the square root of immersion time (in $h^{0.5}$) during hydrothermal treatment at 65 °C for (**a**) GF-PC and (**b**) pure PC specimens. Data for three specimens are presented in different colours.

Figure 5 summarizes the weight gain of the pure PC and GF-PC specimens as functions of time during the hydrothermal treatment, with Figure 5a,b displaying the data for the GF-PC and pure PC specimens, respectively. The curves indicate that the specimen weight increased initially, and reached a plateau after about 100 h, similar to the behaviour reported in ref. [30]. After the weight gain in Figure 5 was normalized by the initial dry weight—defined as $\frac{w-w_{0}}{w_{0}}$, where *w* and $w_{0}$ are the weight of the specimen after and before, respectively, the hydrothermal treatment—an average value of approximately 0.4% was obtained for both pure PC and GF-PC specimens.

**Figure 6:**
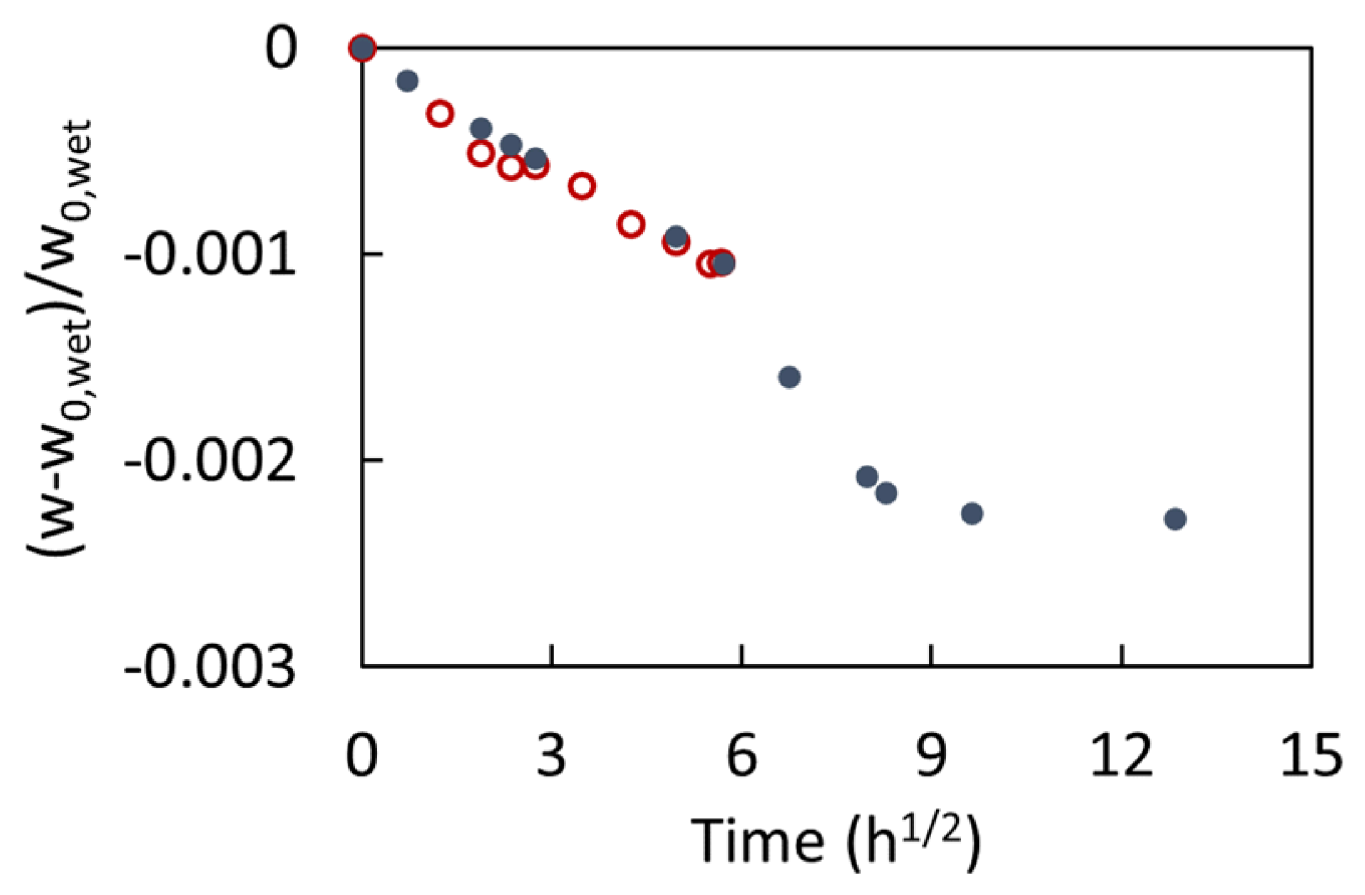
Normalized weight loss in air as a function of time after the hydrothermal treatment for pure PC (filled blue markers) and GF-PC (unfilled red markers) specimens.

Figure 6 presents the normalized moisture loss, defined as $\left(w-w_{0,wet}\right)⁄\left(w_{0,wet}\right)$ where $w_{0,wet}$ represents the initial weight after the hydrothermal treatment. The figure shows that moisture loss during the MR test was similar between GF-PC and pure PC specimens. Both exhibited a mass reduction of around 0.1% by the time at the end of tests of the GF-PC specimens. Therefore, within the timeframe considered in this analysis, i.e., for a period of about 27 h or for nine load–relaxation cycles in the MR test, the specimens still retained a majority of the absorbed water.

### 3.2. MR Test Results

**Figure 7:**
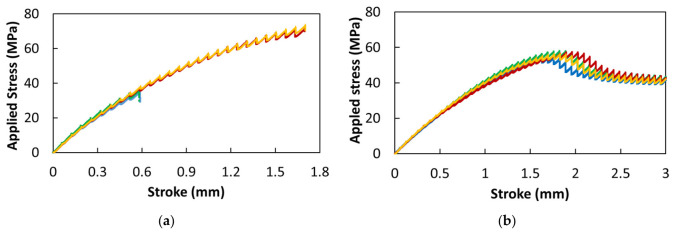
Applied stress–stroke curves from the MR tests on (**a**) GF-PC and (**b**) pure PC specimens. Red and orange curves represent specimens without the hydrothermal treatment, and blue and green curves represent specimens with the hydrothermal treatment.

Figure 7 summarizes curves of applied stress versus stroke from all specimens used in this study, with Figure 7a for GF-PC and Figure 7b for pure PC. Each figure includes specimens with the hydrothermal treatment (blue and green) and without (red and orange). For GF-PC, Figure 7a, the untreated specimens fractured at a stroke around 1.7 mm, while the treated specimens fractured at a much smaller stroke of about 0.58 mm. This suggests that the hydrothermal treatment used in this study could significantly reduce the ductility of GF-PC, causing them to fracture before reaching a yield point. Figure 7a also indicates that the hydrothermal treatment eliminated the effectiveness of the short fibre.

**Figure 8:**
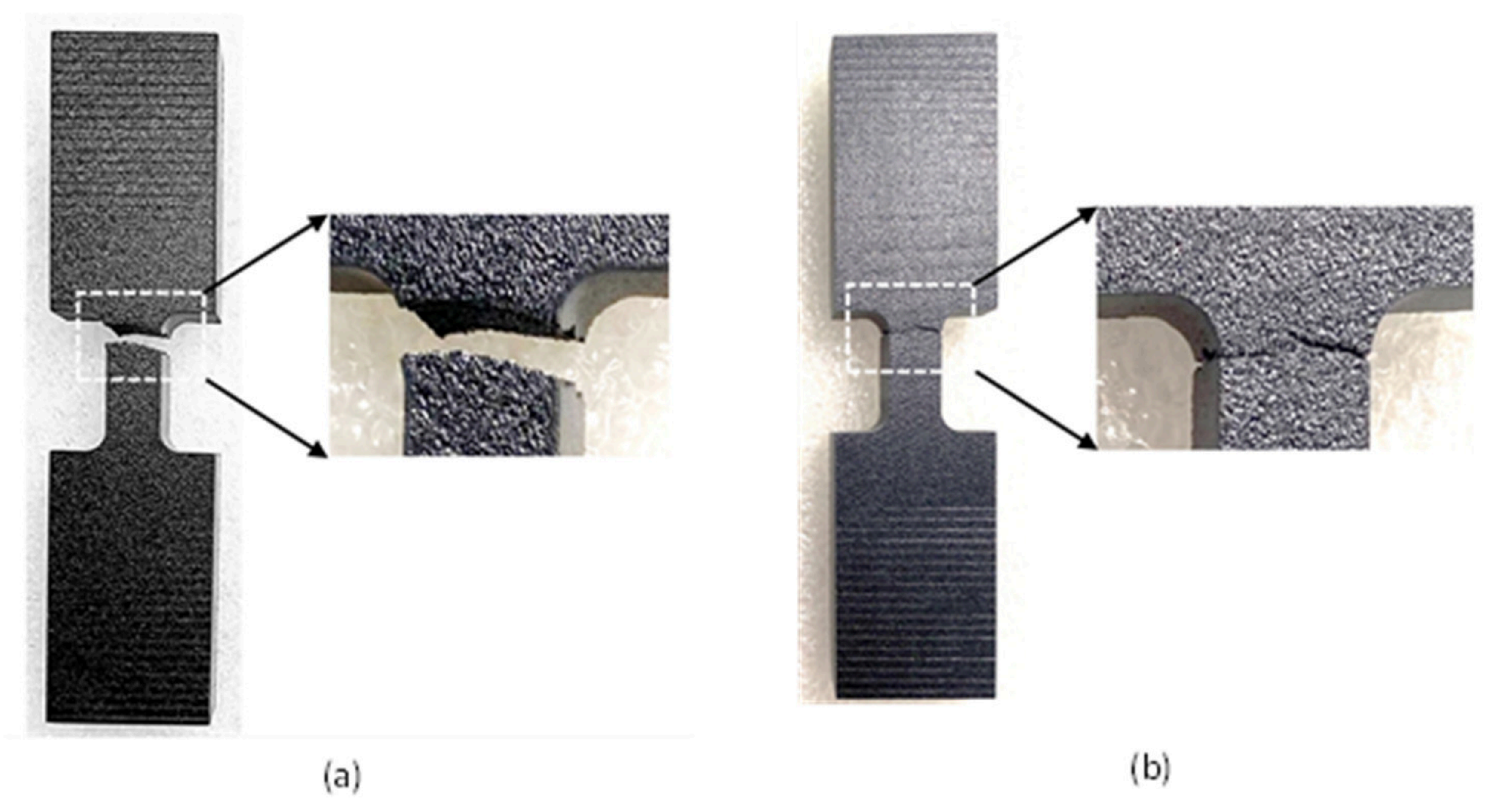
Photographs of GF-PC specimens after the MR test: (**a**) untreated, showing complete separation, and (**b**) hydrothermally treated, showing connection through the gap. For each panel, the dashed box in the left photograph is shown in detail on the right.

It should be pointed out that the untreated GF-PC specimens underwent complete separation into two pieces by the end of the test, as shown in Figure 8a, but the hydrothermally treated specimens did not fracture into two separated halves. Rather, after the machine detected a sudden force drop and automatically stopped, a gap was generated in the specimen, as shown in Figure 8b, and two halves of the specimen remained connected through the gap. However, it only took a force of less than 10% of the maximum force used in the MR test to completely separate the two halves. It is clear that at the end of the test on the hydrothermally treated GF-PC specimens, a small group of glass fibres were responsible for bridging the two halves of the specimens, though the exact mechanisms that led to such a behaviour are not clear and need further investigation.

### 3.3. Stresses Determined from Three- and Four-Branch Spring-Dashpot Models

**Figure 9:**
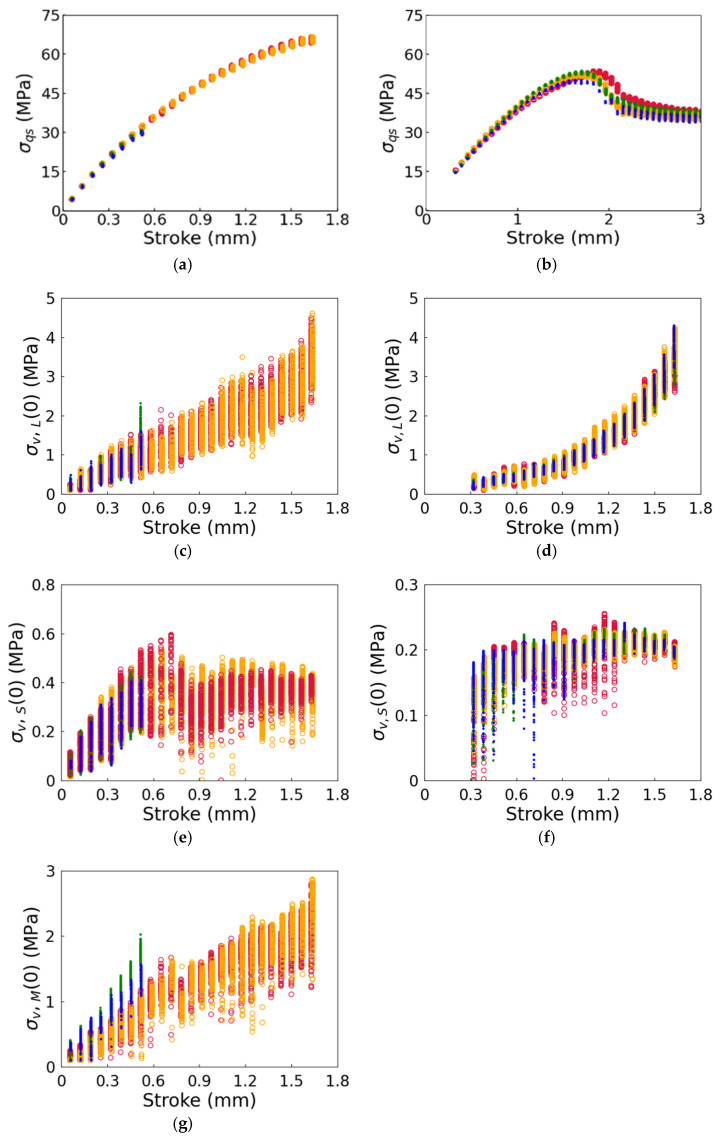
Summary of the 100 sets of values for: (**a**,**b**) $\sigma_{qs}$, (**c**,**d**) $\sigma_{v,L}\left(0\right)$, (**e**,**f**) $\sigma_{v,S}\left(0\right)$, and (**g**) $\sigma_{v,M}\left(0\right)$, with the left column for GF-PC specimens and the right column for pure PC specimens. Red and orange markers represent specimens without the hydrothermal treatment, while blue and green markers represent specimens with the hydrothermal treatment.

**Figure 10:**
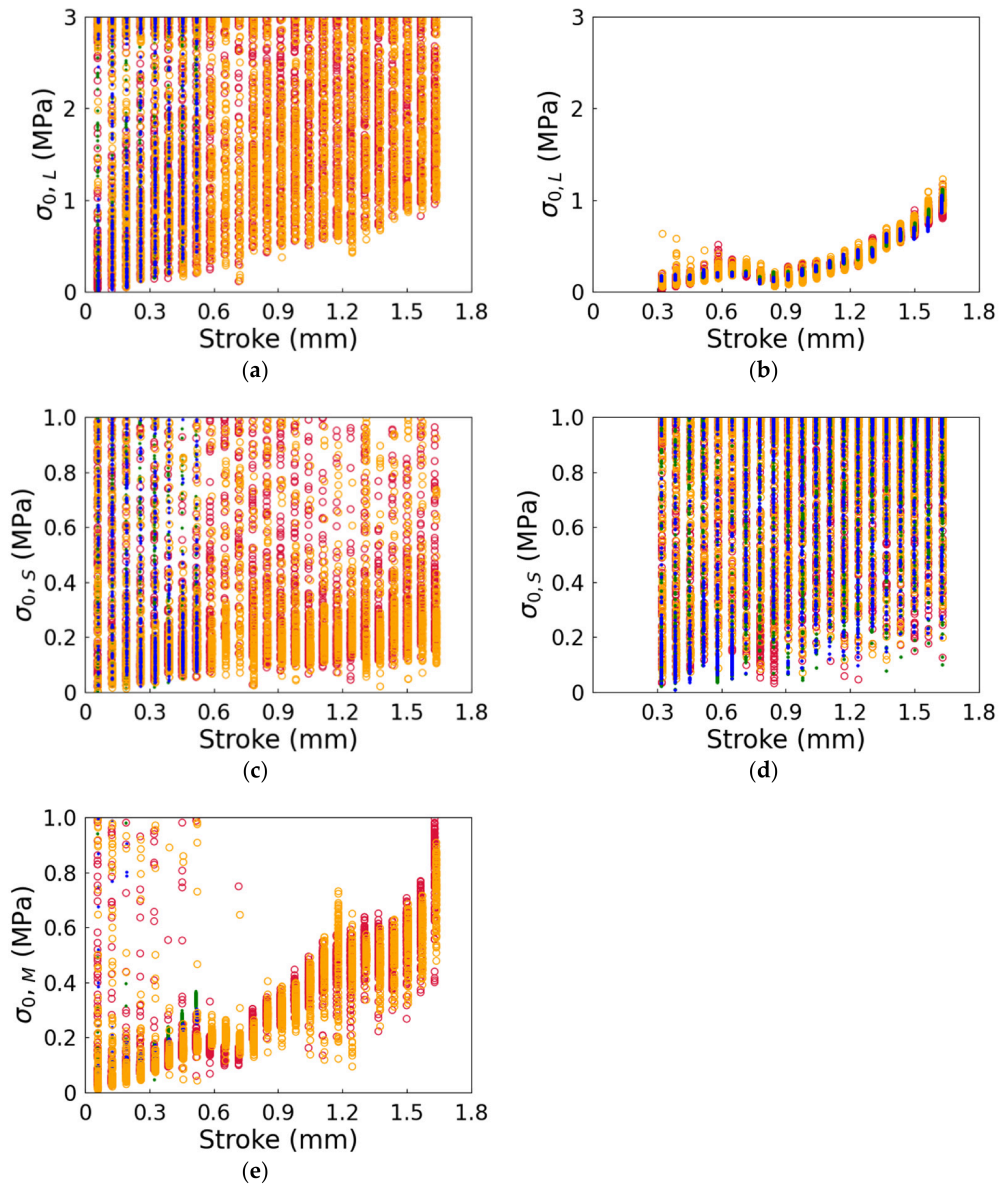
Summary of the 100 sets of values for ***σ***_**0**,**i**_ (i = L, M, S): (**a**,**b**) ***σ***_**0**,**L**_, (**c**,**d**) ***σ***_**0**,**S**_ and (**e**) ***σ***_**0**,**M**_, with the left column showing GF-PC specimens and the right column showing pure PC specimens. Red and orange markers represent specimens without the hydrothermal treatment, while blue and green markers represent specimens with the hydrothermal treatment.

**Figure 11:**
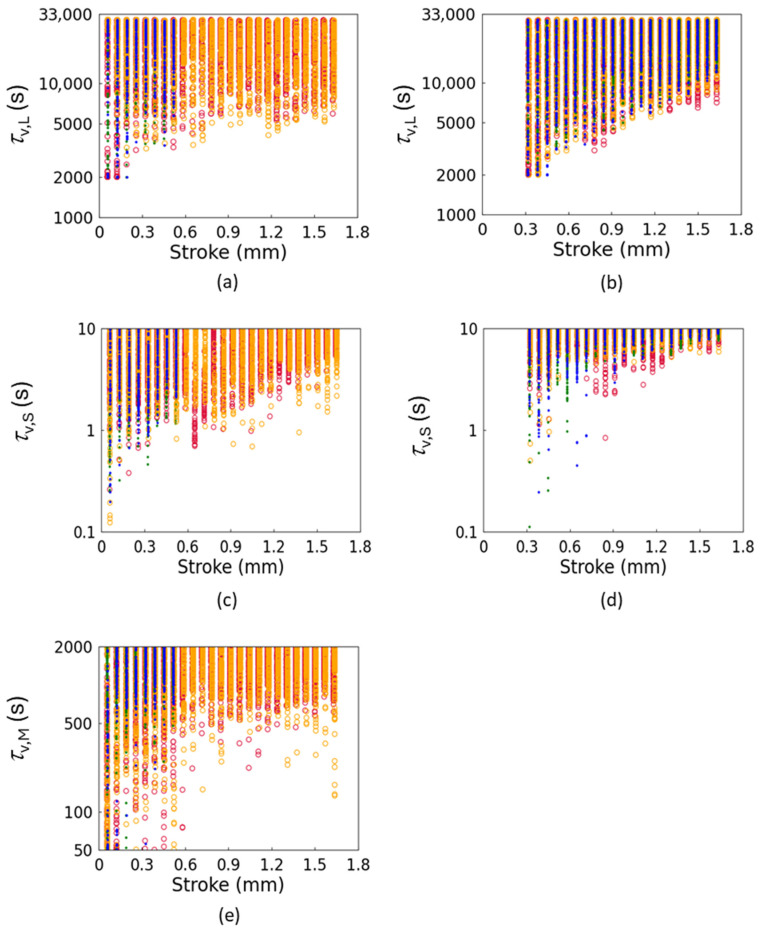
Summary of the 100 sets of values for $\tau_{v,i}$ (i = L, M, S): (**a**,**b**) ***τ***_**0**,**L**_, (**c**,**d**) ***τ***_**0**,**S**_ and (**e**) ***τ***_**0**,**M**_, with the left column showing GF-PC specimens and right column showing pure PC specimens. Red and orange markers represent specimens without the hydrothermal treatment, while blue and green markers represent specimens with the hydrothermal treatment.

The 100 sets of values for $\sigma_{qs}$ and $\sigma_{v,i}\left(0\right)$—where i = L, S for the pure PC specimens and i = L, M, S for the GF-PC specimens—are summarized in Figure 9; the parameters $\sigma_{0,i}$ and $\tau_{v,i}$ used in the spring-dashpot models to replicate stress decay curves at the relaxation stage are summarized in Figure 10 and Figure 11, respectively. To facilitate direct comparison of the same parameters between GF-PC and pure PC, the plots in these figures are arranged in such a way that the plots for the GF-PC data are in the left column of the figures and those for the pure PC data in the right column. Again, red and orange markers represent untreated specimens, while blue and green markers represent the hydrothermally treated specimens. Note that the plots for $\sigma_{v,M}\left(0\right)$, $\sigma_{0,M}$, and $\tau_{v,M}$ appear only in the left column, as the M-branch was used only for the analysis of the GF-PC specimens. Additionally, because the stress decay curves of the pure PC specimens at strokes below 0.3 mm exhibited irregular decreases before the end of the relaxation stages, the corresponding plots in the right column only present values above this stroke threshold. This omission, however, does not hinder the comparison of the variation trends with the corresponding values for the GF-PC specimens.

As shown in Figure 9a,b, for $\sigma_{qs}$ of GF-PC and pure PC, respectively, the hydrothermal treatment yielded little change in the initial rate of increase with the stroke increase. Figure 9b further demonstrates that for pure PC, the peak $\sigma_{qs}$ value was little affected by the hydrothermal treatment. Notably, the $\sigma_{qs}$ values in Figure 9b are presented up to a stroke of 3 mm to illustrate the negligible change of $\sigma_{qs}$ after the necking process started.

The influence of the hydrothermal treatment on the viscous stress ($\sigma_{v,i}\left(0\right)$) is summarized in Figure 9c–g. In summary, the hydrothermal treatment produced no noticeable change, with the sole exception for $\sigma_{v,L}\left(0\right)$ for one GF-PC specimen, at the stroke just prior to the end of the test, as indicated in green in Figure 9c. This abrupt increase in $\sigma_{v,L}\left(0\right)$ value is likely related to localized material instability as fracture was about to occur at the following loading stage. It should be pointed out that this sudden surge of $\sigma_{v,L}\left(0\right)$ did not cause noticeable changes in the trend of variation for $\sigma_{qs}$, as the $\sigma_{qs}$ value is much bigger than the corresponding $\sigma_{v,L}\left(0\right)$ value. Furthermore, the data from the untreated specimens reveal that $\sigma_{v,L}\left(0\right)$ values for GF-PC and pure PC specimens near the stroke of 1.7 mm are remarkably similar, suggesting that for the untreated GF-PC specimens, at the stroke close to the end of the test, $\sigma_{v,L}\left(0\right)$ is insensitive to the presence of glass fibre, in spite that the corresponding $\sigma_{qs}$ values for GF-PC are clearly higher than those for pure PC. On the other hand, at strokes smaller than 1.2 mm, $\sigma_{v,L}\left(0\right)$ values for the GF-PC are slightly higher than those for the pure PC.

In contrast, Figure 9e,f suggest that at a given stroke, $\sigma_{v,S}\left(0\right)$ for GF-PC are clearly higher than that for pure PC, indicating the influence of glass fibre on its value.

Figure 9 also suggests that although the hydrothermal treatment did not affect the trend of increase for $\sigma_{qs}$, $\sigma_{v,L}\left(0\right)$ and $\sigma_{v,S}\left(0\right)$ for both GF-PC and pure PC specimens, the treatment resulted in a significant decrease in ductility for GF-PC and thus prevented these values from reaching their peak levels observed in the untreated specimens. For the pure PC specimens, on the other hand, the hydrothermal treatment exerted no discernible influence on values for these parameters up to the stroke of 1.7 mm.

Unlike $\sigma_{qs}$, $\sigma_{v,L}\left(0\right)$ and $\sigma_{v,S}\left(0\right)$, the $\sigma_{v,M}\left(0\right)$ values for the GF-PC specimens increase after the hydrothermal treatment, as shown in Figure 9g. This increase is somewhat unexpected and is believed to be related to the bridging generated between the two halves of the post-test specimens, as depicted in Figure 8b. This relationship is further discussed in the following section.

## 4. Discussion

The work presented here demonstrates that by employing a multi-branch spring-dashpot model to replicate the relaxation behaviour during MR testing, the stress response of pure PC and GF-PC specimens can be effectively decoupled into a quasi-static component and a viscous counterpart. The results demonstrated that for pure PC, the hydrothermal treatment at 65 °C produced no detectable change in either stress component. Conversely, the hydrothermal treatment increased the viscous stress of the GF-PC specimens, an effect uniquely captured by the M-branch of the model. Given that the moisture absorption of pure PC was comparable to that of GF-PC, and that the hydrothermal treatment was not likely to alter the mechanical response of the unreinforced matrix, the increase in stress response in the M-branch for GF-PC cannot be attributed to the PC matrix itself. Instead, we believe that this viscous stress increase reflects the influence of the hydrothermal treatment on the fibre–matrix interface.

We also hypothesize that the M-branch is needed to replicate the relaxation behaviour of the GF-PC in the MR test because deformation of the PC matrix surrounding the fibre–matrix interface could be affected by the hydrothermal treatment. As the interface is expected to swell due to the water absorption, additional stress could be applied to its surrounding matrix, which leads to the change in the viscous deformation behaviour. Although this concept is merely a speculation at this stage, it has some support from Figure 9g in which $\sigma_{v,M}\left(0\right)$ shows increase after the hydrothermal treatment. Another support comes from the value ranges for reference stress ($\sigma_{0}$) and characteristic relaxation time ($\tau_{v}$) which, as shown in Figure 10 for $\sigma_{0}$ and Figure 11 for $\tau_{v}$, are different among the three viscous branches. This leads us to wonder whether the microstructural region represented by the M-branch could be different from those represented by the L- and S-branches, though we are still looking for the evidence to support this speculation.

## 5. Conclusions

Immersion in water at 65 °C was used to investigate the influence of the hydrothermal treatment on the mechanical behaviour of GF-PC, determined using the MR test results, through the analysis based on spring-dashpot models. The study provides some evidence to indicate that the hydrothermal treatment could cause a significant loss in the ductility, without changing the initial specimen stiffness, as indicated in Figure 7a.

By employing a four-branch spring-dashpot model to decouple the quasi-static stress from the viscous stress components, the study found that the initial stiffness for the quasi-static stress component did not change, either. However, due to the loss of the ductility, the maximum quasi-static stress for GF-PC was significantly reduced by the hydrothermal treatment.

The stress responses of GF-PC from the three viscous branches of the spring-dashpot model showed some differences in the trend of change after the hydrothermal treatment. For the L- and S-branches, the stress–stroke relationship is not affected by the hydrothermal treatment; while for the M-branch a clear increase for the viscous stress was found after the hydrothermal treatment.

For the pure PC specimens, none of the above changes were detected after the hydrothermal treatment. Although the material characteristics for the pure PC and PC matrix in GF-PC may not be similar, and thus the comparison of results between GF-PC and pure PC could not be quantified at this stage, the results do suggest that the hydrothermal treatment could only give a very limited change in PC. In view that the glass fibre should not be affected by the hydrothermal treatment, it is reasonable to attribute the changes detected in GF-PC to the change at the fibre–matrix interface where water could be absorbed during the hydrothermal treatment. The interconnection between the two halves of GF-PC specimens after the MR test provides some support to this speculation, but a further study is needed to obtain solid evidence. Nevertheless, the results show that fibre may not be as effective in the reinforcement of PC matrix after the exposure to water. The study shows that the proposed approach of using MR testing and spring-dashpot modelling could be used to characterize the change in the mechanical properties and thus could be used as a new means to characterize the efficacy of fibre reinforcement on polymers.

## Figures and Tables

**Table 1:** Search scopes for parameters in the three-branch model for pure PC specimens.

$\sigma_{v,S}\left(0\right)$ (MPa)	$\sigma_{0,S}$ (MPa)	$\tau_{v,S}$ (s)	$\sigma_{v,L}\left(0\right)$ (MPa)	$\sigma_{0,L}$ (MPa)	$\tau_{v,L}$ (s)
[0, 2]	[0, 1]	[0, 10]	[0, 20]	[0, 3]	[2000, 3000]

**Table 2:** Search scopes for parameters in the four-branch model for GF-PC specimens.

$\sigma_{v,S}\left(0\right)$ (MPa)	$\sigma_{0,S}$ (MPa)	$\tau_{v,S}$ (s)	$\sigma_{v,M}\left(0\right)$ (MPa)	$\sigma_{0,M}$ (MPa)	$\tau_{v,M}$ (s)	$\sigma_{v,M}\left(0\right)$ (MPa)	$\sigma_{0,L}$ (MPa)	$\tau_{v,L}$ (s)
[0, 2]	[0, 1]	[0, 10]	[0, 20]	[0, 1]	[50, 2000]	[0, 20]	[0, 3]	[2000, 30,000]

## Data Availability

The original contributions presented in this study are included in the article. Further inquiries can be directed to the corresponding author.

## Abbreviations

The following abbreviations are used in this manuscript:

GF-PC	Short glass fibre reinforced polycarbonate
MR	Multi-relaxation
PC	Polycarbonate
${\dot{\delta }}_{d,i}$	Stroke rate of the dashpot in branch *i* of the spring-dashpot model
${\dot{\delta }}_{0,i}$	Reference stroke rate of branch *i* in a spring-dashpot model
$K_{v,i}$	Spring modulus in viscous branch *i* of the spring-dashpot model
$\Delta \sigma_{v}\left(t\right)$	Total stress decay of a spring-dashpot model
$\sigma_{0,i}$	Reference stress of branch *i* in a spring-dashpot model
$\sigma_{A}$	Applied stress in the MR test
$\sigma_{qs}$	Quasi-static stress
$\sigma_{v,i}\left(t\right)$	Viscous stress of branch *i* as a function of time *t* from the onset of the relaxation phase, *i* = *L*, *M* and *S*
$\tau_{v,i}$	Characteristic relaxation time in viscous branch *i* of the spring-dashpot model
*w*	Measured weight of the specimen
*w*_0_	Initial weight before the hydrothermal treatment
